# Multi-omics identification of MBNL2 associated with poor pathological response to neoadjuvant chemoimmunotherapy in lung squamous cell carcinoma

**DOI:** 10.3389/fonc.2026.1877829

**Published:** 2026-08-19

**Authors:** Hao Wu, Yang Cheng, Honglin Yan, Shiying Zhang, Hang Chen, Haochen Xue, Honglei Chen, Jingping Yuan

**Affiliations:** 1 Department of Pathology, Renmin Hospital of Wuhan University, Wuhan, Hubei, China; 2 School of Basic Medical Sciences, Wuhan University, Wuhan, Hubei, China; 3 Clinical Laboratory, General Hospital of the Central Theatre Command, Wuhan, Hubei, China; 4 Department of Pathology, Zhongnan Hospital of Wuhan University, Wuhan, Hubei, China

**Keywords:** cancer-associated fibroblasts, immunotherapy response, lung squamous cell carcinoma, MBNL2, prognostic model, regulatory T cells, tumor microenvironment

## Abstract

**Background:**

Neoadjuvant chemoimmunotherapy (NACI) improves outcomes in resectable lung squamous cell carcinoma (LUSC), yet response varies widely and current biomarkers lack precision. Novel correlates of immunotherapy sensitivity tailored to the LUSC tumor microenvironment (TME) are urgently needed.

**Methods:**

Using TCGA-LUSC transcriptomic data, we constructed a 25-gene prognostic model and applied three machine learning algorithms in combination with the Tumor Immune Dysfunction and Exclusion (TIDE) algorithm to identify core genes linked to prognosis and immunotherapy response. Immune infiltration and enrichment analyses were performed to characterize the TME. An independent pre-NACI biopsy cohort (n=36) was used for histopathological validation to explore correlations with pathological response, while single-cell RNA-seq (GSE207422) and CellChat were used to infer tumor-stromal crosstalk and explore underlying mechanisms.

**Results:**

The risk score independently stratified prognosis. Among three core genes, high MBNL2 expression was associated with higher TIDE scores, lower TIDE-predicted response rates, and elevated cancer-associated fibroblast (CAF) scores. scRNA-seq revealed systematically enhanced communication between MBNL2-high tumor cells and FAP^+^ CAFs, with unique ligand-receptor pairs enriched in WNT and EGF pathways; FAP^+^ CAFs interacted with regulatory T cells via ECM remodeling and the MDK-NCL axis. Histopathological validation confirmed that low tumor-cell MBNL2 expression correlated with higher pathological response and pCR rates, reduced FAP, and decreased FOXP3 expression.

**Conclusion:**

This study establishes a 25-gene prognostic model for LUSC and identifies MBNL2 as a novel correlate of poor pathological response to NACI. Elevated MBNL2 expression in tumor cells is associated with enhanced tumor-CAF crosstalk, CAF activation, and an immunosuppressive TME, laying a foundation for future mechanistic investigation.

## 1 Introduction

Despite remarkable advances in early diagnosis and comprehensive treatment of lung cancer in recent years, its incidence and mortality rates have remained persistently high, posing a severe global health challenge (1). Lung squamous cell carcinoma (LUSC) is a major histological subtype of non-small cell lung cancer (NSCLC), accounting for approximately 20-30% of all lung cancer cases (2). In lung adenocarcinomas (LUAD), targeted therapy, particularly interventions targeting receptor tyrosine kinases, has achieved remarkable success, driving the exploration of similar strategies in LUSC. However, driver mutations that are common and highly targetable in LUAD are extremely rare in LUSC, and screenings for LUSC-specific driver mutations have also failed to yield breakthrough discoveries (3).

Recently, immunotherapy represented by immune checkpoint inhibitors (ICIs), particularly neoadjuvant chemoimmunotherapy based on programmed death-1 (PD-1)/programmed death-ligand 1 (PD-L1) inhibitors, has significantly improved event-free survival (EFS) and pathological complete response (pCR) in some patients with advanced or resectable NSCLC (4). In neoadjuvant chemoimmunotherapy (NACI) studies, pCR (defined as 0% residual viable tumor (RVT)) and major pathological response (MPR; ≤10% RVT) have emerged as reliable surrogate endpoints for evaluating treatment efficacy in place of EFS, owing to their objectivity and early measurability. Non-major pathological response (Non-MPR) is defined as the presence of >10% RVT, representing insufficient pathological regression. Incorporating this classification into the evaluation framework enables a nuanced assessment of therapeutic response and facilitates the identification of patient subgroups that derive varying degrees of benefit from NACI (5).

Unfortunately, most patients with LUSC present at an advanced stage at initial diagnosis, with limited systemic treatment options available, and even after receiving NACI, marked heterogeneity of treatment response remains substantial (3, 6). In LUSC, PD-L1 expression and tumor mutational burden (TMB) — core biomarkers associated with immunotherapeutic response — exhibit limited discriminative accuracy and translational utility, with current evidence failing to fully reconcile the exigencies of precise patient stratification. While PD-L1 tumor proportion score (TPS) remains the predominant correlative metric, its association with durable benefit is not absolute: a subset of TPS ≥50% patients derive no sustained advantage, whereas meta-analyses document survival gains even among TPS <1% cohorts (7–10). Relying solely on this biomarker may therefore overlook potential responders. The clinical utility of TMB, in turn, is constrained by the lack of assay standardization and the absence of uniform cutoffs (11–13). Critically, durable responses to ICIs have been observed in some patients with low TMB, whereas primary resistance occurs in a subset of patients with high TMB—a discordance that substantially undermines its standalone utility as a stratifying biomarker (14, 15). Thus, there remains an unmet and pressing need to identify novel, alternative correlates of immunotherapeutic sensitivity specifically tailored to the LUSC context. Notably, multi-gene expression signatures combined with tumor microenvironment characterization have recently been employed to develop prognostic models and predict immunotherapy response in hepatocellular carcinoma, ovarian cancer, and lung adenocarcinoma (16–19). These studies demonstrate the utility of such integrative approaches across diverse cancer types and provide a methodological foundation for similar investigation in LUSC.

The tumor microenvironment (TME) is central to tumor initiation, progression, and therapeutic resistance, in which the complex interplay between immune and stromal cells, particularly cancer-associated fibroblasts (CAFs), critically shapes an immunosuppressive state and modulates immunotherapeutic response (20–22). Research indicates that CAFs expressing fibroblast activation protein (FAP) can promote immune evasion through various mechanisms, such as secreting immunosuppressive cytokines, remodeling the extracellular matrix (ECM), and recruiting other immunosuppressive cells like regulatory T cells (Tregs), and are associated with primary resistance to ICIs (23–27). Therefore, systematically characterizing the TME features of LUSC, particularly identifying key molecules that reflect the functional status of CAFs and their communication with immune cells, is of great value for understanding the mechanisms of immunotherapy resistance and developing novel stratifying tools.

In this study, we sought to systematically characterize the TME of LUSC and identify novel biomarkers associated with response to NACI. By integrating TCGA transcriptomic data, we constructed a 25-gene prognostic model and, using three machine learning algorithms combined with the Tumor Immune Dysfunction and Exclusion (TIDE) algorithm, further pinpointed muscleblind-like protein 2 (MBNL2) as a core gene linked to poor prognosis and an immunosuppressive TME. Histopathological validation in an independent pre-NACI biopsy cohort confirmed that higher MBNL2 expression in tumor cells correlated with poor pathological response to NACI, while single-cell RNA-seq analysis revealed potential tumor-CAF crosstalk, CAF activation, and features of an immunosuppressive microenvironment, laying a foundation for future mechanistic investigation.

## 2 Materials and methods

### 2.1 Patient validation cohort and sample collection

A retrospective analysis was performed on pathological archives from Renmin Hospital of Wuhan University (March 2022–March 2025). A total of 36 patients with LUSC who received 1–4 cycles of NACI followed by surgical resection, and had complete postoperative pathological response data, were included (**Table 1**). All cases were initially diagnosed as LUSC via lung puncture or bronchoscopic biopsy at Renmin Hospital of Wuhan University before surgery, and postoperative specimens were uniformly reviewed by thoracic subspecialty pathologists according to current pathological diagnostic criteria.

**Table 1:** Clinical parameters of 36 patients receiving NACI.

Characteristics		Non-response	Response	
		Non-MPR	MPR	pCR
Age (y)	<60	4	2	4
	≥60	11	8	7
Gender	Male	14	10	10
	Female	1	0	1
Diameter of tumor (cm)	< 4	5	4	2
	≥4	10	6	9
Number of treatment cycles	1	1	1	0
	2	6	6	9
	3	4	3	2
	4	4	0	0

### 2.2 Data acquisition and preprocessing

The transcriptome data of LUSC were obtained from The Cancer Genome Atlas (TCGA) via the Xena browser (https://xenabrowser.net/datapages/). Raw RNA-seq data (STAR count and FPKM) were processed using R 4.5.0. Samples were filtered to retain primary tumors (01A) and adjacent normal tissues (11A). The FPKM matrix was integrated with protein-coding gene annotations, normalized, and deduplicated by gene symbol, yielding three final matrices (all samples, tumor only, normal only) for downstream analysis.

### 2.3 Differential gene expression analysis

Differential expression analysis between tumor and normal samples was performed using the DESeq2 R package with a negative binomial generalized linear model. Genes satisfying |log_2_fold change| > 1 and a false discovery rate (FDR)-adjusted P-value (padj) < 0.05 were considered significantly differentially expressed genes (DEGs).

### 2.4 Univariate and multivariate Cox proportional hazards regression analysis

Clinical survival data of LUSC patients were obtained from TCGA, and overall survival (OS) time and status were extracted. The processed tumor-only FPKM expression data were matched with survival information, retaining samples with both profiles. Univariate Cox regression was performed for each differentially expressed gene to preliminarily screen prognostic genes. Subsequently, multivariate Cox regression was applied to the candidate genes to evaluate their independent predictive value, thereby identifying key prognostic genes.

### 2.5 Least absolute shrinkage and selection operator Cox regression model construction

To construct a robust prognostic signature and avoid overfitting, LASSO-Cox regression was performed using the R package glmnet on prognosis-related genes identified from prior univariate and multivariate Cox analyses. The optimal penalty parameter λ (λ.min) was determined by 10-fold cross-validation, and genes with non-zero coefficients at λ.min were retained. A risk score for each patient was calculated based on gene expression levels and their coefficients (risk score = Σ gene expression × coefficient). Patients were stratified into high- and low-risk groups using the median risk score as the cutoff.

### 2.6 Assessment of predictive performance

The receiver operating characteristic (ROC) curve and area under the curve (AUC) for factors such as the risk score in relation to survival status were calculated. Time-dependent ROC analysis was used to evaluate the predictive performance of the model at 1-year, 3-year, and 5-year time points, implemented with the time ROC package.

### 2.7 Survival analysis

Survival curves for high-risk and low-risk groups (or high- and low-expression groups of specific genes) were plotted using the Kaplan-Meier method, and the log-rank test was used to compare survival differences between the two groups. Visualization was performed using the R package survminer.

### 2.8 Nomogram development and validation

Clinical data for the TCGA-LUSC cohort were downloaded from the Xena database (https://xenabrowser.net/datapages/) and integrated with the previously calculated risk scores. Samples with complete clinical information, survival data, and risk scores were retained. A nomogram was constructed using a multivariable Cox regression model incorporating daily smoking consumption, age, pathological grade, and risk score to predict 1-, 3-, and 5-year survival probabilities. Model discrimination was assessed by AUC at the same time points, and internal validation was performed using bootstrap resampling with calibration curves to evaluate concordance between predicted and observed probabilities.

### 2.9 Assessment of immunotherapy response

The TIDE algorithm was employed to evaluate the potential clinical response to ICIs in the TCGA -LUSC cohort. TIDE scores and associated immune signatures were obtained from the Harvard Dana-Farber online platform (https://cide.ccr.cancer.gov). TCGA-LUSC samples were stratified into high- and low-expression groups based on the median mRNA level of target genes, and TIDE-predicted response rates and immune signatures were compared between subgroups.

### 2.10 Correlation of LASSO-derived signature with TIDE immune landscape

The collective enrichment of the LASSO-derived prognostic gene signature was quantified using single-sample Gene Set Enrichment Analysis (ssGSEA) on the LUSC cohort expression matrix, yielding a LASSO signature GSVA score for each sample. Pearson correlation analysis was then conducted to evaluate the association between this score and multiple TIDE-derived immune metrics. The association between the final prognostic risk score and the same immune parameters was also assessed. Correlation matrices were generated, with statistical significance set at adjusted P < 0.05.

### 2.11 Validation of gene importance based on machine learning

To assess the robustness of the LASSO-Cox-derived gene signature and identify core driver genes, feature importance analysis was performed using three machine learning algorithms: gradient boosting machine (GBM), random forest (RF), and support vector machine (SVM). For each algorithm, a model was built using survival status and expression levels of the selected genes. Model performance was evaluated by AUC with cross-validation. For GBM, hyperparameters were optimized through grid search by examining combinations of n.trees, interaction.depth, shrinkage, and n.minobsinnode, with shrinkage fixed at 0.1 and n.minobsinnode fixed at 10; the optimal combination that maximized AUC was n.trees = 50, interaction.depth = 2, shrinkage = 0.1, n.minobsinnode = 10. For SVM with a radial basis kernel, tuning was performed via 10-fold cross-validation using caret. In the grid generated by tuneLength = 5, the optimal hyperparameters that maximized AUC were sigma = 0.03196471 and C = 0.25. For RF, a forest with 1,000 trees was built using the randomForest package, and the number of variables randomly sampled at each split was set to the default square root of the number of predictors. Feature importance was measured by the algorithm-specific metric: mean decrease in Gini for RF, relative influence based on loss reduction for GBM, and permutation importance (via caret’s varImp) for SVM. For each algorithm, genes with importance above the median were defined as high-contribution genes.

### 2.12 ssGSEA immune cell infiltration analysis

TCGA-LUSC samples were stratified by risk group (high vs. low), and ssGSEA was performed using the GSVA R package to estimate enrichment scores for 28 immune cell types per sample.

### 2.13 Analysis of immune checkpoint gene expression

Expression levels of 12 key immune checkpoint genes (PD-1/PDCD1, PD-L1/CD274, PD-L2/PDCD1LG2, CTLA-4, LAG-3, TIM-3/HAVCR2, TIGIT, VISTA/VSIR, ICOS, OX40/TNFRSF4, GITR/TNFRSF18, and B7-H3/CD276) were extracted from the expression matrix and compared between high- and low-risk groups (or between high- and low-expression groups of specific genes). The selection of these genes was based on their well-established roles in immune checkpoint pathways and their relevance to cancer immunotherapy (28–34).

### 2.14 ESTIMATE immune score analysis

The ESTIMATE algorithm was applied to TCGA-LUSC samples stratified by high- and low-expression of the target gene, yielding StromalScore, ImmuneScore, ESTIMATE Score, and TumorPurity. Grouping information was matched with ESTIMATE scores, and appropriate statistical tests were selected based on data distribution and variance homogeneity.

### 2.15 Functional enrichment analysis

Samples were dichotomized into high- and low-expression groups based on the target gene’s median expression across all samples. Differential expression analysis was performed using the DESeq2 package, with genes meeting the criteria of adjusted p-value (padj) < 0.05 and |log_2_fold change| > 1 defined as significantly DEGs. To investigate the potential biological functions of the identified DEGs, Gene Ontology (GO) enrichment analysis, encompassing biological process (BP), cellular component (CC), and molecular function (MF) terms, was conducted using the org.Hs.eg.db database and the clusterProfiler package. Subsequently, Kyoto Encyclopedia of Genes and Genomes (KEGG) pathway enrichment analysis was performed to identify the key signaling pathways and biological processes associated with the DEGs.

### 2.16 Single-cell RNA sequencing data analysis

#### 2.16.1 Single-cell data preprocessing and clustering

The scRNA-seq UMI count matrix and corresponding metadata from the GSE207422 dataset were loaded to construct a Seurat object. Cells with fewer than 200 or more than 8,000 detected genes, as well as those with mitochondrial gene content > 20% or hemoglobin gene content > 3%, were excluded. After filtering, the data were normalized using the LogNormalize method, and the top 2,000 highly variable genes were selected for principal component analysis (PCA). Cell clustering was performed using the first 15 principal components with a resolution of 0.6, followed by uniform manifold approximation and projection (UMAP) for visualization.

#### 2.16.2 Cell type annotation and identification of malignant epithelial cells

Major cell lineages were annotated based on the expression of canonical markers: PTPRC and CD3E for T cells, CD79A, MS4A1, and IGHG1 for B and plasma cells, LYZ and CSF3R for myeloid cells and neutrophils, KIT for mast cells, VWF for endothelial cells, COL1A1 for fibroblasts, EPCAM for epithelial cells, and MKI67 for proliferating cells. To refine the T-cell compartment, T cells were re-clustered and further annotated into subtypes using established markers: CD4 for CD4^+^ T cells, CD8A and CD8B for CD8^+^ T cells, FOXP3 and IL2RA for Tregs, FGFBP2 and FCGR3A for natural killer cells, and MKI67 for proliferating T cells. These refined T-cell subtypes were incorporated into the complete Seurat object. To distinguish malignant cells from normal epithelial cells within the epithelial compartment, the CopyKAT algorithm was applied, classifying individual cells as aneuploid (malignant) or diploid (normal) based on genome-wide copy number profiles inferred from scRNA-seq data.

#### 2.16.3 Cell-cell communication analysis with CellChat

CellChat was applied to infer ligand-receptor interactions. For the tumor to FAP^+^ CAF axis, shared ligand-receptor pairs were compared between MBNL2-high and MBNL2-low groups using the Wilcoxon paired rank-sum test and visualized with paired line plots, while group-specific pairs were displayed using pathway-stratified dot plots. For the FAP^+^ CAF to Treg axis, significant pairs (P < 0.05) were visualized in bubble plots.

### 2.17 Immunohistochemistry staining

Tissue specimens were fixed, dehydrated, and paraffin-embedded. Immunohistochemistry was performed following standard protocols. After heat-induced epitope retrieval, sections were incubated with primary antibodies: mouse anti-human MBNL2 (1:400, Santa Cruz, 3B4), rabbit anti-human FAP (1:2000, HUABIO, JA56-11), and rabbit anti-human FOXP3 (1:400, Cell Signaling Technology, D6O8R), followed by horseradish peroxidase-conjugated secondary antibodies and hematoxylin counterstaining. Whole-slide scanning was performed using a KFSlideOS system under a 20× objective. MBNL2 staining in preoperative biopsy specimens was evaluated by a blinded thoracic pathologist who delineated tumor areas with reference to H&E-stained sections. Due to the limited size of the biopsy tissue, all tumor areas were delineated on the MBNL2-stained slides. Subsequently, quantitative analysis of all demarcated tumor regions was performed using the IHC Profiler plugin in ImageJ software. An H-score was calculated according to the standard formula: H-score = Σ (PI × i) = (percentage of weak staining × 1) + (percentage of moderate staining × 2) + (percentage of strong staining × 3). Finally, the arithmetic mean of the H-scores was calculated and used as the final MBNL2 expression level for each case in subsequent statistical analyses. Patients were divided into high- and low-expression groups based on the median. FAP and FOXP3 staining were evaluated similarly.

### 2.18 Multiplex immunofluorescence staining

Multiplex immunofluorescence staining was performed using the Kreep™ 7-Color Kit (KR Pharmtech, Shanghai, China) according to the manufacturer’s protocol. Formalin-fixed, paraffin-embedded LUSC sections were dewaxed, rehydrated, and subjected to antigen retrieval with Tris-EDTA buffer (pH 9.0). After blocking, sections were incubated with primary antibodies against CD138 (1:1, DAKO, MI15), FAP (1:2000, HUABIO, JA56-11), MBNL2 (1:400, Santa Cruz, 3B4), FOXP3 (1:400, Cell Signaling Technology, D6O8R), and Pan-CK (1:100, Fuzhou Maixin, AE1/AE3). Each primary antibody was visualized using tyramide signal amplification with kit-provided fluorophores, followed by DAPI counterstaining. Whole-slide scanning was performed at 20 × using the KR-HT5 system, and data were analyzed with inForm 2.4.0 software. A spectral library was created for autofluorescence subtraction and cell segmentation (nuclear, membrane, cytoplasmic) using built-in artificial intelligence. Target cell populations were identified by mean fluorescence intensity, and the algorithm was applied to all samples.

### 2.19 Statistical analysis

All statistical analyses were performed using R software. For two-group comparisons, Student’s t-test (normal, equal variance), Welch’s t-test (normal, unequal variance), or Wilcoxon rank-sum test (non-normal) was applied as appropriate. The Benjamini-Hochberg method was used for FDR control when necessary. For multi-group comparisons, one-way analysis of variance (parametric) or Kruskal-Wallis test (non-parametric) was used. Significance levels: “***” for P < 0.001, “**” for P < 0.01, “*” for P < 0.05, and “ns” for P ≥ 0.05. For histopathological validation, patients were stratified into MBNL2-high and -low groups based on the median H-score. The median was chosen as a pre-specified, unbiased cutoff to maintain balanced group sizes for statistical comparisons in this relatively small cohort (n=36), to avoid overfitting a data-driven optimal threshold derived from ROC analysis, and to ensure consistency with the median-based dichotomization strategy uniformly applied across all preceding TCGA transcriptomic analyses.

## 3 Results

### 3.1 Construction of a prognostic model for LUSC

**Figure 1:**
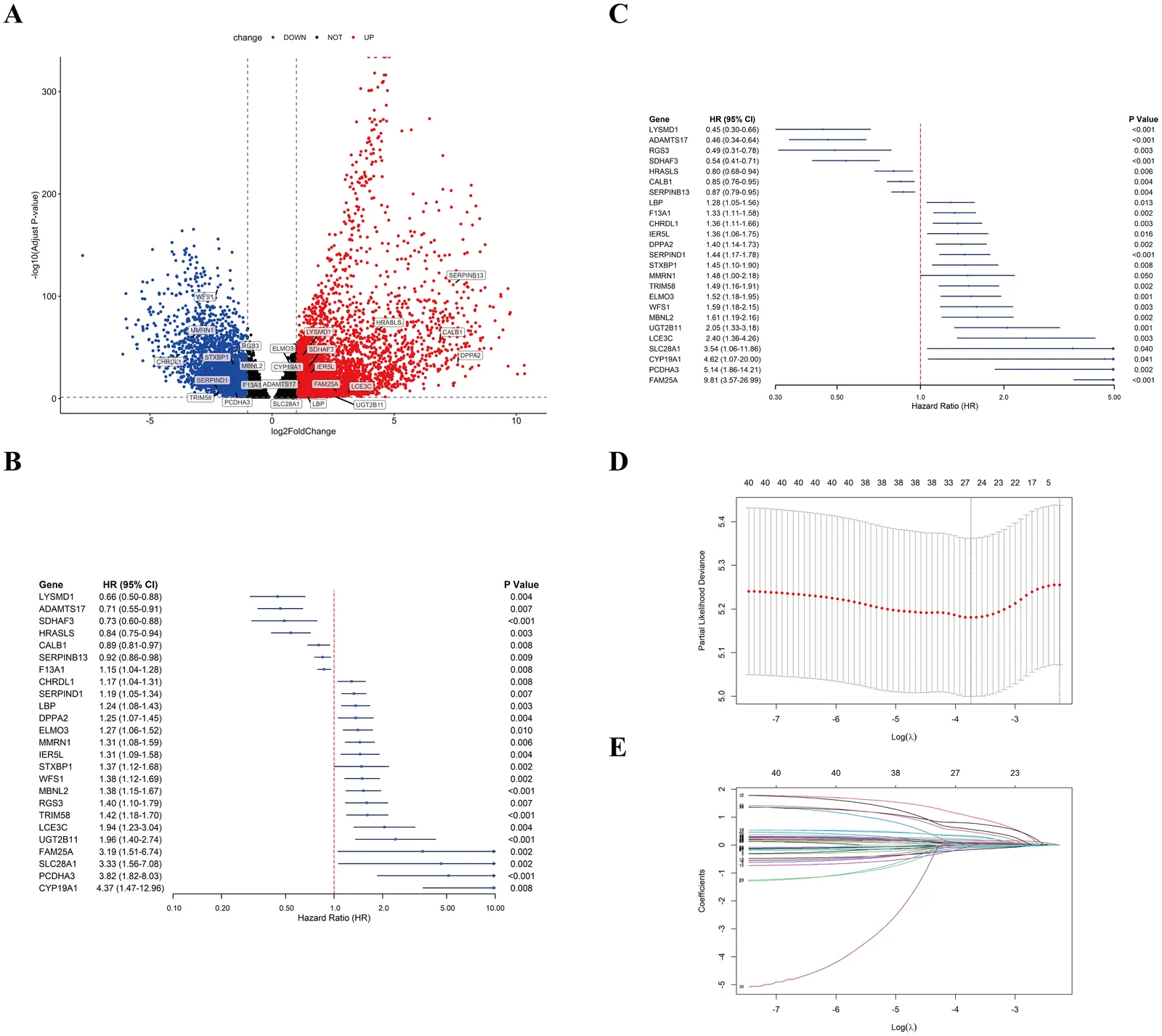
Construction of a prognostic risk model in the TCGA cohort. **(A)** The volcano map shows differential gene expression between normal and LUSC tumor tissues. We use red dots to indicate upregulated genes in tumor tissue and blue dots to show down-regulated genes in tumor tissue. **(B)** Univariate Cox regression analysis of differentially expressed genes (Forest plot of the LASSO genes). **(C)** Multivariable Cox regression analysis (Forest plot of the LASSO genes). **(D)** The best log Lambda value was selected for the TCGA cohort through 10-fold cross-validation in the LASSO regression model. **(E)** LASSO coefficient plot.

The workflow for constructing and analyzing the prognostic model for LUSC is illustrated in **Supplementary Figure 1**. From 496 LUSC tumor and 51 normal tissues in the TCGA cohort, a total of 7,159 DEGs were identified (**Figure 1A**). Univariate Cox regression screened out 152 prognosis-associated DEGs (**Figure 1B**), and multivariate Cox analysis further refined 43 genes (**Figure 1C**). To construct a robust prognostic signature and avoid overfitting, we performed LASSO Cox regression analysis and ultimately selected 25 genes to build the prognostic risk model (**Figures 1D, E**). The risk score was calculated as: Risk score = -0.161896 × ADAMTS17 + 1.021727 × FAM25A +… + 0.029544 × MMRN1 (**Supplementary Table 1**).

### 3.2 Risk model validation

**Figure 2:**
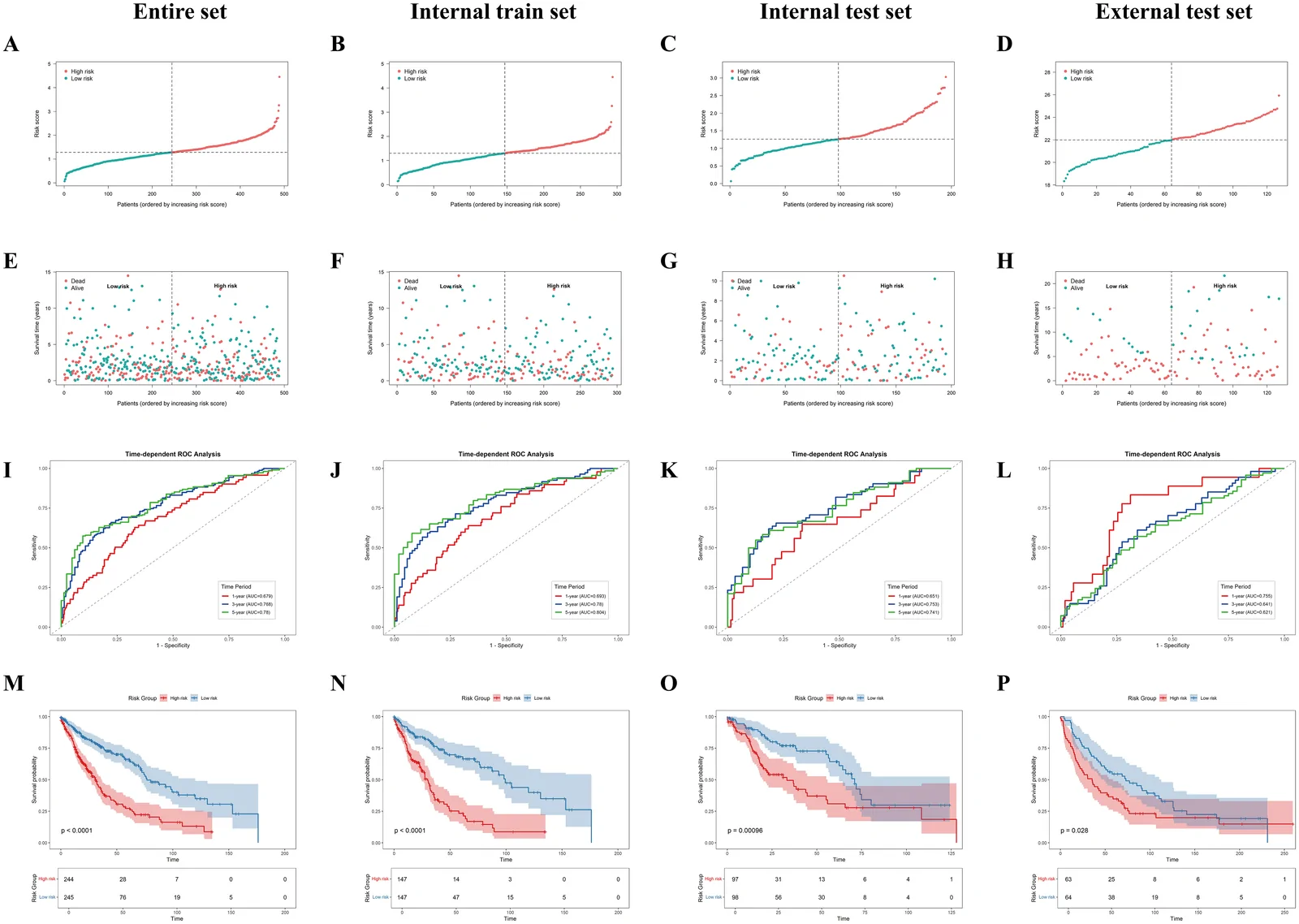
Validation of the LASSO prognostic model in the entire, train, test set, and GEO cohort. **(A-D)** Exhibition of predictive model based on risk score of the entire train, test set, and GEO cohort, respectively. **(E-H)** Survival time and survival status between low-risk and high-risk groups in the entire train, test set, and GEO cohort, respectively. **(I-L)** Time-dependent ROC curves of 1-, 3-, and 5-year of LUSC patients in the entire, train, test set, and GEO cohort, respectively. **(M-P)** Kaplan-Meier survival curves for OS between low-risk and high-risk groups in the entire, train, and test sets, and in the GEO cohort, respectively.

To assess model stability, 489 LUSC patients with survival information from the TCGA cohort (full cohort) were randomly divided into a training set (n=294) and a testing set (n=195) at a 6:4 ratio for internal validation. For external validation, two independent GEO cohorts (GSE37745 and GSE30219) were integrated. To eliminate cross-platform expression bias, batch effect correction was performed using the ComBat function from the sva R package. The expression matrix was first restricted to genes common to both datasets. The dataset of origin was treated as the batch variable, and an intercept-only model matrix (model.matrix(~1, data = data.frame(batch = batch))) was specified to preserve biological variability unrelated to the batch. The ComBat function was applied with the following parameters: par.prior = TRUE (parametric prior estimation for robust adjustment in small cohorts) and prior.plots = FALSE. The effectiveness of batch correction was assessed by comparing the proportion of variance attributable to the batch variable before and after adjustment. Risk score distribution (**Figures 2A–D**), survival status (**Figures 2E–H**), ROC curves (**Figures 2I–L**), and survival outcomes (**Figures 2M–P**) were compared across the full cohort, training set, testing set, and external set. No significant discrepancies were observed among the four groups. Risk curves and scatter plots showed a positive correlation between mortality and risk scores. ROC curves demonstrated favorable predictive accuracy, and Kaplan-Meier analysis indicated significantly better overall survival in low-risk groups across all four cohorts (P < 0.0001, P < 0.0001, P = 0.00096, and P = 0.028, respectively). Thus, the risk score serves as a reliable prognostic marker for LUSC.

### 3.3 Evaluation of the risk model as an independent prognostic factor

**Figure 3:**
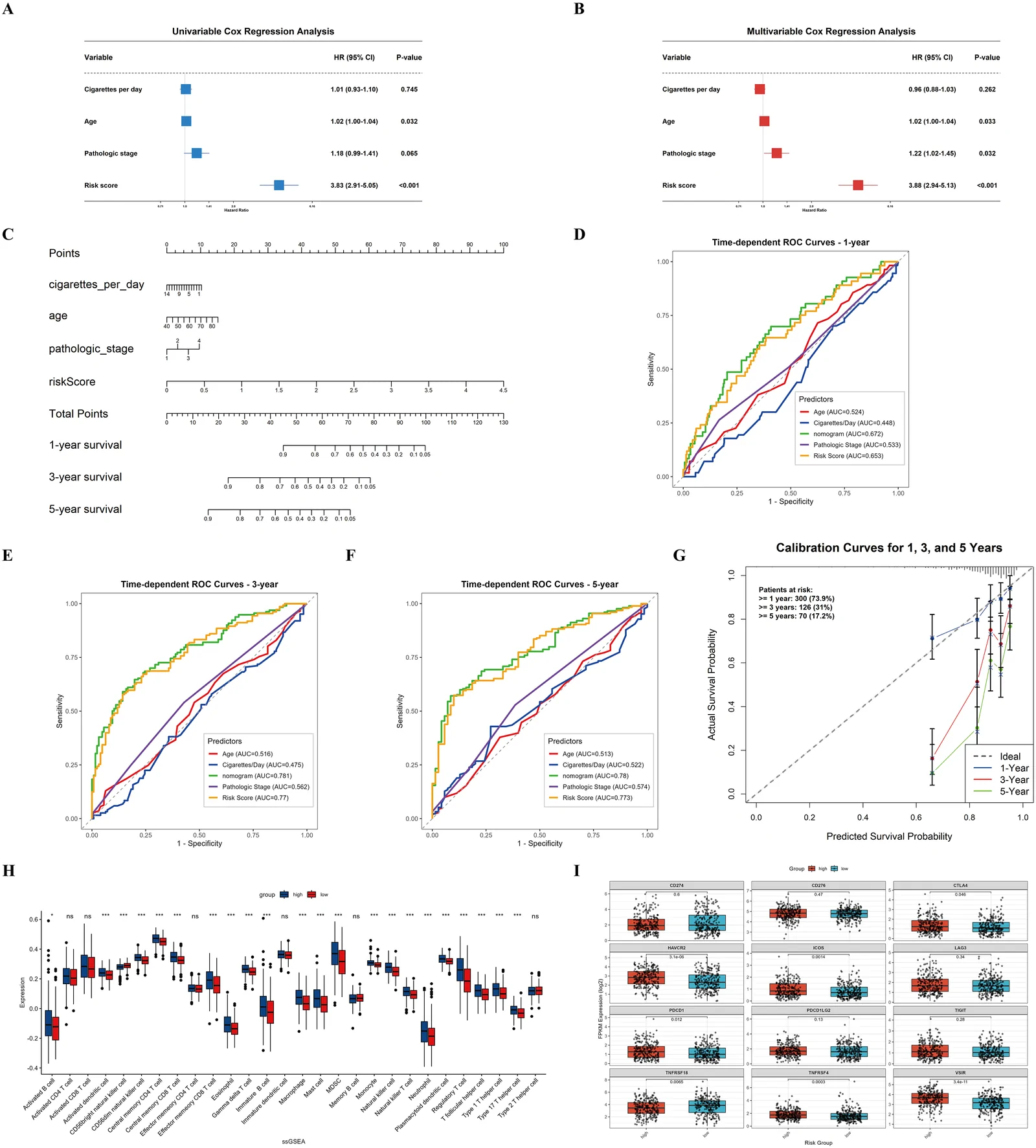
Assessment of the risk model as an independent prognostic factor and analysis of immunological factors. **(A, B)** The univariate and multivariate Cox regression analysis of risk score and clinical features regarding prognostic value. **(C)** A clinical prognostic nomogram was developed to predict 1-, 3-, and 5-year survival. **(D-F)** Time-dependent ROC curve analyses for predicting OS by risk and clinical features. **(G)** The calibration curve was used to evaluate the predictive accuracy of the nomogram. **(H)** ssGESA analysis of differences in 28 types of immune cell infiltration in the LUSC tumor microenvironment between high-risk and low-risk groups. **(I)** The expression of 12 immune checkpoints differs between the high-risk and low-risk groups. *P-value* < 0.05 indicates statistical significance. *P < 0.05; **P < 0.01; ***P < 0.001; ns, non-significant.

Univariate and multivariate Cox regression analyses showed that the risk score was an independent prognostic factor for LUSC (hazard ratio (HR) = 3.83 and 3.88, respectively; both P < 0.001; **Figures 3A, B**). A nomogram integrating daily smoking amount, age, pathological grade, and risk score was constructed to predict 1-, 3-, and 5-year survival (**Figure 3C**). The AUC of the nomogram at 1, 3, and 5 years was 0.672, 0.781, and 0.780, respectively, while the risk score alone yielded AUCs of 0.653, 0.770, and 0.773, showing superior performance to other clinical factors (**Figures 3D–F**). Calibration curves showed good agreement at 1 year, but slight overestimation at 3 and 5 years (**Figure 3G**). This progressive deviation may be attributable to two contributing factors. First, the number of patients at risk decreased substantially during later follow-up, reducing the stability of probability estimates at these time points. Second, the overestimation may reflect potential violations of the Cox proportional hazards assumption, which requires that the hazard ratio between risk groups remains constant over time. If the prognostic effect of certain covariates diminishes with prolonged follow-up — a phenomenon commonly observed in cancer survival models — the predicted survival probabilities at later time points would overestimate the actual survival. Nonetheless, the model’ s consistently high AUC at all time points (0.781 and 0.780 at 3 and 5 years, respectively) supports its robust discriminative ability, indicating that it effectively distinguishes between risk groups even if absolute probability calibration at later time points is imperfect. Accordingly, the nomogram should be regarded primarily as a reliable risk-stratification tool rather than a precise predictor of absolute survival probabilities at extended follow-up. Overall, the risk score and nomogram appear promising for prognostic stratification in LUSC; however, given their derivation from retrospective data, external prospective validation is required before clinical application.

### 3.4 Immune infiltration analysis of the risk model

ssGSEA revealed significant differences in immune cell infiltration between high- and low-risk groups. The high-risk group showed higher abundances of activated B cells (P < 0.05), activated dendritic cells, macrophages, mast cells, myeloid-derived suppressor cells (MDSCs), NK cells, neutrophils, pDCs, and Tregs (all P < 0.001), while no significant differences were observed for activated CD4^+^ or CD8^+^ T cells (**Figure 3H**). Analysis of 12 immune checkpoint genes (**Figure 3I**) showed that PDCD1, CTLA4, HAVCR2, ICOS, TNFRSF4, and VSIR were highly enriched in the high-risk group, whereas TNFRSF18 was significantly higher in the low-risk group.

### 3.5 Correlation of LASSO-derived features with TIDE immune indicators

**Figure 4:**
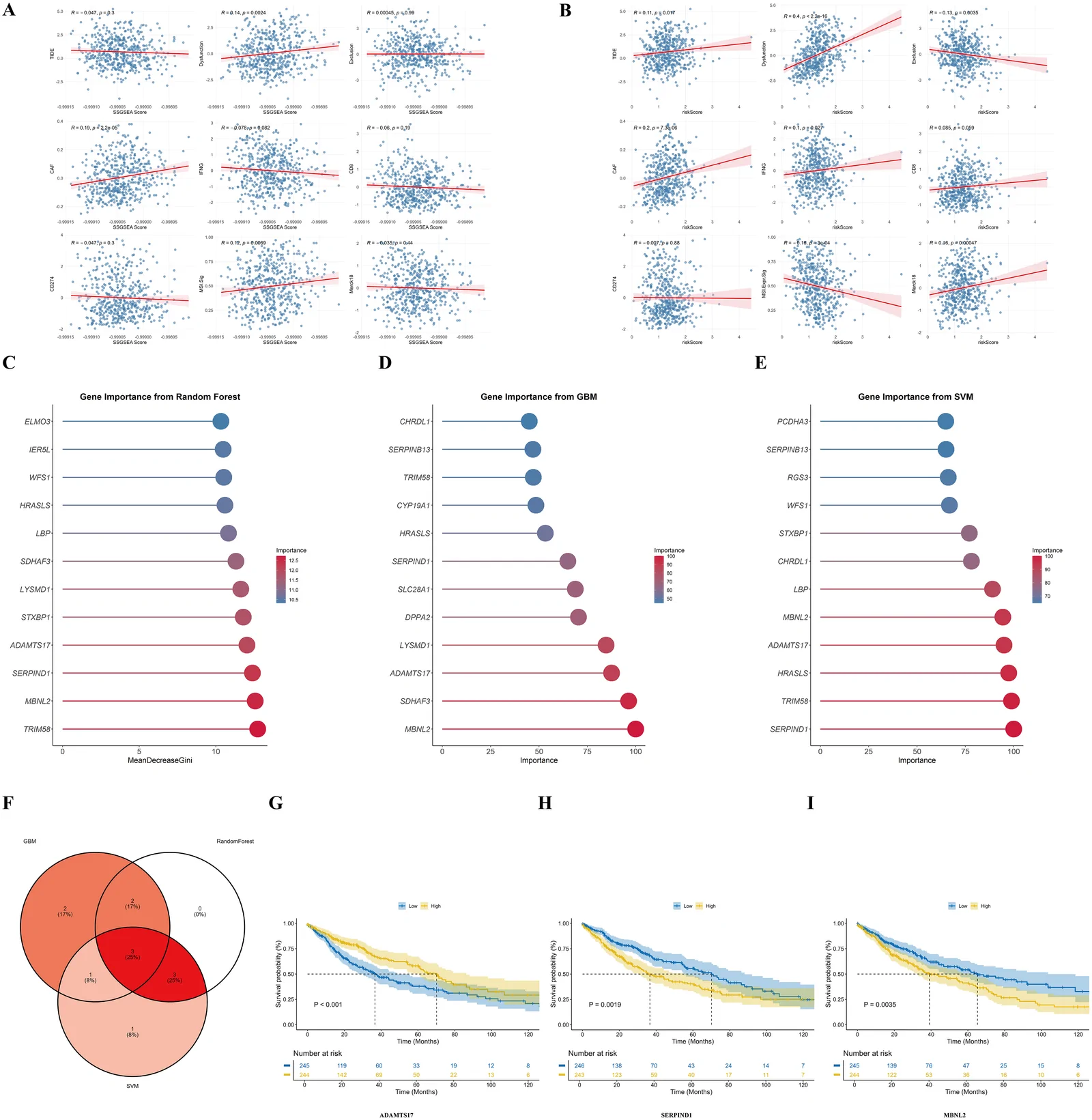
Correlation analysis, feature importance, and survival outcomes of the LASSO-derived signature. **(A)** Pearson correlations between the LASSO signature ssGSEA score and TIDE-derived immune metrics. **(B)** Pearson correlations between the riskScore and TIDE-derived immune metrics. **(C)** Random forest algorithm for assessing gene importance in the risk model. **(D)** The GBM algorithm for assessing gene importance in the risk model. **(E)**The SVM algorithm for assessing gene importance in the risk model. **(F)** Intersection of three machine learning algorithms via venn diagram. **(G-I)** Kaplan-Meier survival curves of OS between high- and low-expression groups of ADAMTS17, SERPIND1, and MBNL2, respectively.

Pearson correlation analysis revealed that both the LASSO signature ssGSEA score and the risk score were significantly associated with multiple TIDE-derived immune metrics in the TCGA-LUSC cohort. The ssGSEA score was positively correlated with CAF (r = 0.189, P < 0.001), Dysfunction (r = 0.136, P < 0.01), and MSI.Expr.Sig (r = 0.121, P < 0.01) (**Figure 4A**). The risk score showed positive correlations with Dysfunction (r = 0.396, P < 0.001), CAF (r = 0.201, P < 0.001), Merck18 (r = 0.158, P < 0.001), TIDE (r = 0.108, P < 0.05), and IFNG (r = 0.100, P < 0.05), and negative correlations with MSI.Expr.Sig (r = -0.163, P < 0.001), Exclusion (r = -0.132, P < 0.01). No significant correlations were observed with CD8 or CD274 for either feature (both P > 0.05) (**Figure 4B**).

### 3.6 Evaluating the importance of genes in the risk model using machine learning algorithms

Three machine learning algorithms (RF, GBM, and SVM) were used to assess the feature importance of the risk model genes. For each algorithm, genes with importance scores above the median were defined as high-contribution genes (**Figures 4C–E**). Intersection analysis of the top eight high-contribution genes from each algorithm yielded three core genes: ADAMTS17, SERPIND1, and MBNL2 (**Figure 4F**). Kaplan-Meier analysis showed that patients with high ADAMTS17 expression had significantly better OS (P < 0.001), while low expression of SERPIND1 and MBNL2 was associated with better survival (P = 0.0019 and P = 0.0035, respectively) (**Figures 4G–I**). All three genes were significantly correlated with survival, verifying the reliability of the machine learning approach.

### 3.7 Assessment of core gene signatures in association with immunotherapy response using the TIDE algorithm

**Figure 5:**
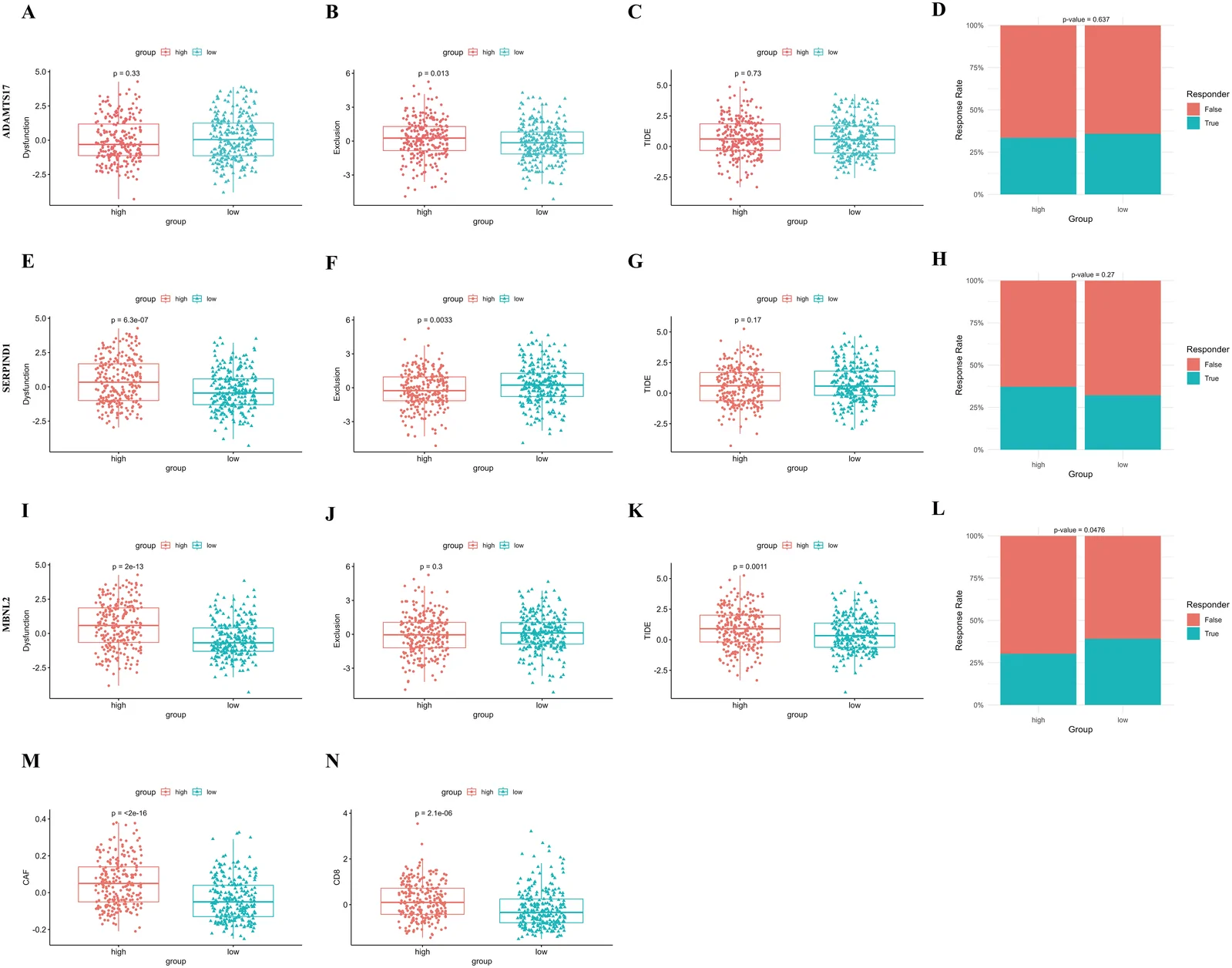
Association of core gene signatures with immunotherapy response assessed via TIDE algorithm. **(A, E, I)** Compare the differences in Dysfunction Scores between high and low expression groups of ADAMTS17, SERPIND1, and MBNL2, respectively. **(B, F, J)** Compare the differences in Exclusion Scores between high and low-expression groups for ADAMTS17, SERPIND1, and MBNL2, respectively. **(C, G, K)** Compare the differences in TIDE Scores between high and low expression groups of ADAMTS17, SERPIND1, and MBNL2, respectively. **(D, H, L)** Compare the Response Rate differences between high- and low-expression groups of ADAMTS17, SERPIND1, and MBNL2, respectively. **(M, N)** Compare the differences in CAF Score and CD8 Score between the high and low expression groups of MBNL2.

TCGA-LUSC samples were divided into high- and low-expression groups based on median expression of ADAMTS17, SERPIND1, and MBNL2. The potential association with immunotherapy response was evaluated using the TIDE algorithm. The SERPIND1-high and MBNL2-high groups showed significantly higher dysfunction scores than their low-expression counterparts, while no difference was observed for ADAMTS17 (**Figures 5A, , I**). Exclusion scores were higher in the ADAMTS17-high group, lower in the SERPIND1-high group, and not different for MBNL2 (**Figures 5B, F, J**). TIDE scores were significantly higher in the MBNL2-high group, with no differences for ADAMTS17 or SERPIND1 (**Figures 5C, G, K**). The TIDE-predicted immunotherapy response rate was significantly higher in the MBNL2-low group (**Figures 5D, H, L**). Additionally, the MBNL2-high group exhibited significantly higher CAF and CD8 scores than the low-expression group (**Figures 5M, N**).

### 3.8 Immune scores and enrichment analysis between high- and low-expression groups of MBNL2

**Figure 6:**
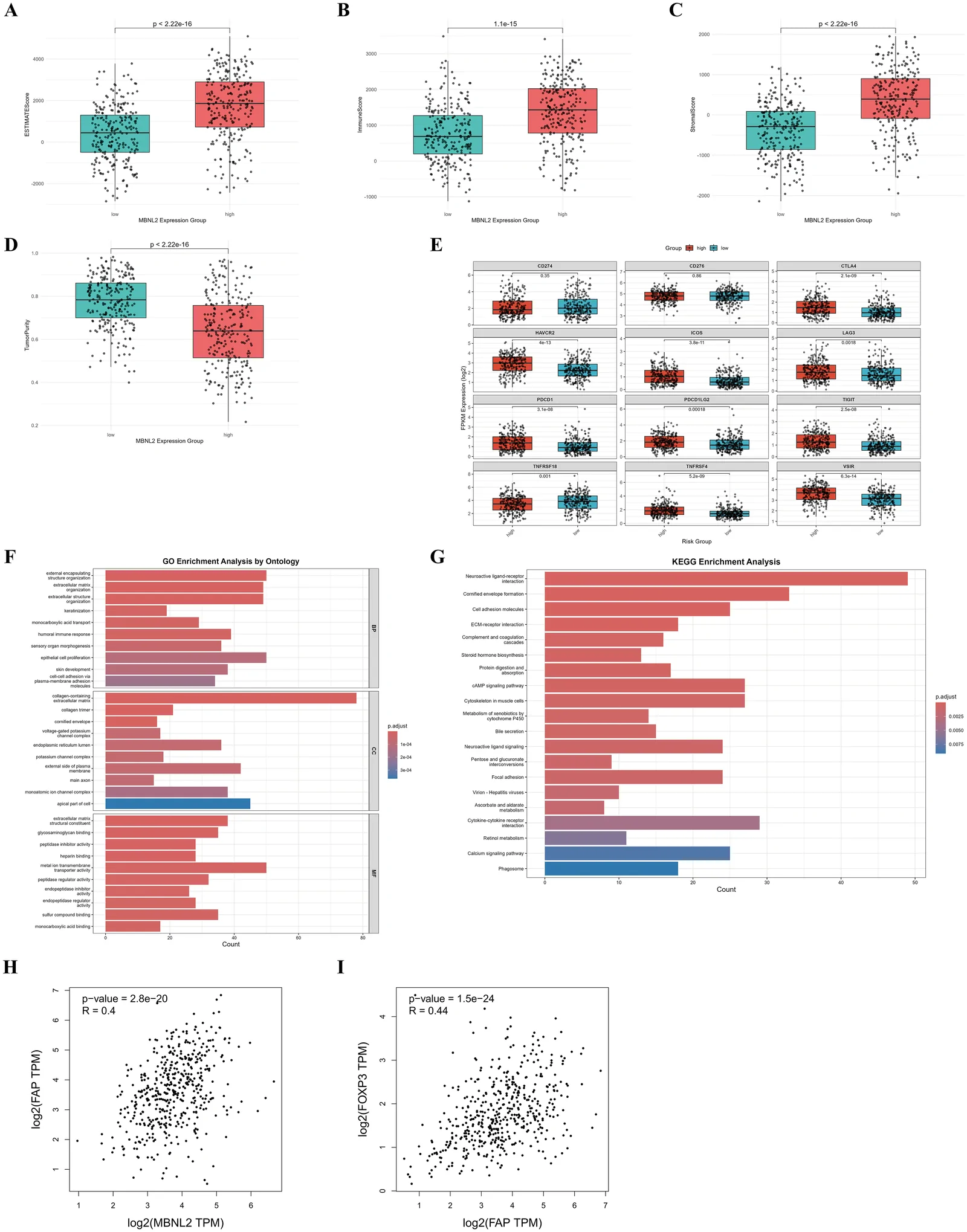
Analyses of immune infiltration and enrichment between high and low MBNL2 expression groups. **(A-D)**Comparison of ESTIMATE score, immune score, stromal score, and tumor purity between high and low MBNL2 expression groups. **(E)** The expression of 12 immune checkpoints differs between the high and low MBNL2 expression groups. **(F, G)** Functional enrichment analysis of Gene Ontology (GO) and pathway enrichment analysis of Kyoto Encyclopedia of Genes and Genomes (KEGG) were performed on differentially expressed genes (DEGs) between high and low MBNL2 expression groups in LUSC patients. **(H, I)** Correlation analysis of expression levels between MBNL2 and FAP, and FAP and FOXP3 in LUSC.

Using the ESTIMATE algorithm, the MBNL2-high group showed significantly higher ESTIMATE, Immune, and Stromal scores, and markedly lower Tumor Purity than the low-expression group (**Figures 6A–D**). Nine immune checkpoint genes (CTLA4, HAVCR2, ICOS, LAG3, PDCD1, PDCD1LG2, TIGIT, TNFRSF4, VSIR) were highly enriched in the MBNL2-high group, while TNFRSF18 was more highly expressed in the low-expression group (**Figure 6E**). Despite higher CD8, ESTIMATE, and Immune scores in the MBNL2-high group, the enrichment of multiple immune checkpoints suggests the occurrence of CD8^+^ T cell exhaustion.

To further explore the potential mechanisms by which MBNL2 regulates prognosis and influences immunotherapy response in LUSC patients, we performed GO functional enrichment analysis and KEGG pathway enrichment analysis based on the differentially expressed genes between the high- and low-expression groups of MBNL2 in LUSC patients. In the GO functional enrichment analysis, MBNL2 was primarily involved in extracellular matrix organization, extracellular structure organization, humoral immune response (BP); collagen-containing extracellular matrix, collagen trimer (CC); and extracellular matrix structural constituent, glycosaminoglycan binding, peptidase inhibitor activity (MF) (**Figure 6F**). The KEGG pathway enrichment analysis results showed that the cell adhesion molecules and ECM-receptor interaction pathways were significantly enriched in the MBNL2-high group (**Figure 6G**). TIDE analysis showed a positive correlation between MBNL2 and CAF score (**Figure 5M**), which is consistent with the enrichment analysis results indicating that MBNL2 is primarily involved in ECM structural constituents, among others. Recent studies have shown that CAFs expressing FAP drive an immunosuppressive TME by recruiting and supporting the survival of CD4^+^ CD25^+^ FOXP3^+^ Tregs and increasing their overall abundance, thereby contributing to primary resistance to immune checkpoint inhibitors (24, 26). To explore the aforementioned mechanism, we used GEPIA (http://gepia.cancer-pku.cn/) to perform Spearman correlation analysis. The results revealed significant positive correlations: MBNL2 with FAP (R = 0.40, P = 2.8×10^-^²^0^), and FAP with FOXP3 (R = 0.44, P = 1.5×10^-^²^4^) (**Figures 6H, I**).

### 3.9 Single-cell RNA sequencing analysis of the GSE207422 dataset

**Figure 7:**
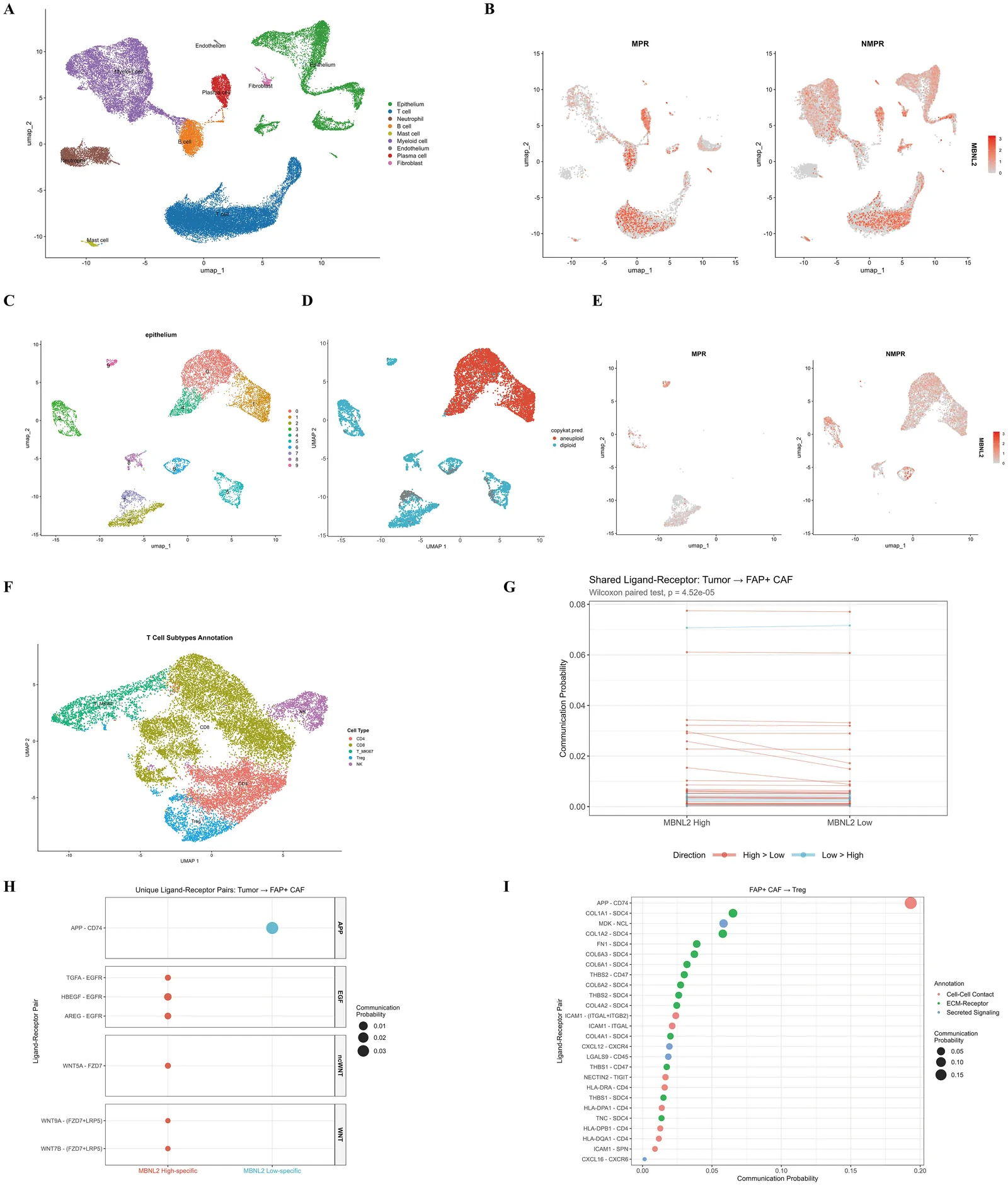
Single-cell transcriptomic landscape of the LUSC microenvironment and cell-cell communication analysis. **(A)** A uniform manifold approximation and projection plot of all cells, colored by major types using canonical markers, annotating them into 9 distinct cell types. **(B)** Split UMAP visualization showing the expression pattern of MBNL2 in the MPR versus NMPR groups at the single-cell level. **(C)** Subclustering analysis reveals transcriptionally distinct epithelial subpopulations. **(D)** Clustering patterns of malignant versus normal epithelial cells. **(E)** Split UMAP comparing MBNL2 expression in epithelial cells between MPR and NMPR groups. Note: One case with unknown pathological response is not shown. **(F)** Annotation of T cell subclusters into CD4^+^ T cells, CD8^+^ T cells, proliferating T cells, regulatory T cells, and NK cells. **(G)** MBNL2-high malignant cells exhibit higher communication probabilities with FAP^+^ CAFs than MBNL2-low cells. **(H)** Unique ligand-receptor pairs in MBNL2-high versus MBNL2-low subgroups along the tumor to FAP^+^ CAF axis, enriched in WNT and EGFR pathways. **(I)** Bubble plot showing significant ligand-receptor pairs along the FAP^+^ CAF to Treg axis.

Single-cell RNA-seq data from LUSC samples following NACI (GSE207422) were processed with quality control, dimensionality reduction, and clustering. Analysis of marker genes (PTPRC, CD3E, LYZ, CD79A, MS4A1, IGHG1, CSF3R, KIT, VWF, COL1A1, EPCAM, MKI67) across 23 clusters (**Supplementary Figure 2A**) allowed for the annotation of nine cell types: T cells, B cells, myeloid cells, neutrophils, plasma cells, mast cells, fibroblasts, endothelial cells, and epithelial cells (**Figure 7A**). Split UMAP visualization further revealed the single-cell expression pattern of MBNL2 between the MPR and NMPR groups (**Figure 7B**). Subsequently, particular attention was focused on MBNL2 expression differences in tumor cells between the two response groups. Epithelial cells were extracted and subjected to subclustering analysis, which revealed multiple transcriptionally distinct subpopulations (**Figure 7C**). Dot plot analysis of top marker genes confirmed the molecular heterogeneity across these subclusters (**Supplementary Figure 2B**). Application of the CopyKAT algorithm distinguished aneuploid (malignant) from diploid (normal) epithelial cells (**Supplementary Figure 2C**), with malignant cells showing distinct clustering patterns (**Figure 7D**). Finally, split UMAP visualization directly compared the distribution of MBNL2 expression in epithelial cells between the MPR and NMPR groups (**Figure 7E**).

Notably, MBNL2 expression in tumor cells varied significantly between the NMPR and MPR groups: NMPR tumors contained a distinct MBNL2-positive subpopulation alongside MBNL2-negative cells, whereas the sparse residual tumor cells captured from MPR specimens were uniformly MBNL2-negative. Although the naturally low abundance of residual tumor cells in MPR tumor beds limits sampling depth, this consistent negativity contrasts sharply with the defined MBNL2-expressing subpopulation observed in NMPR tumors. To further characterize the immune components within the TME, we performed in-depth analysis of the T-cell compartment. Based on expression profiling of lineage-defining genes across the eight T cell subclusters (**Supplementary Figure 2D**) and their upregulated marker genes (**Supplementary Figure 2E**), the subclusters were annotated as CD4^+^ T cells, CD8^+^ T cells, proliferating T cells, regulatory T cells, and NK cells (**Figure 7F**).

To investigate how MBNL2 expression in tumor cells shapes the TME, we focused on the NMPR group due to the limited number of cases in both groups and the scarcity of malignant cells in MPR specimens. NMPR malignant cells were stratified into MBNL2-high and MBNL2-low subgroups based on median expression, and ligand-receptor interactions were inferred using CellChat. Along the tumor to FAP^+^ CAF axis, 44 shared ligand-receptor pairs were identified between the two subgroups. Communication probabilities were systematically higher in the MBNL2-high subgroup than in the MBNL2-low subgroup, with a paired Wilcoxon test revealing a highly significant difference (p < 0.0001, **Figure 7G**). Among these 44 shared pairs, the number of pairs exhibiting higher communication probabilities in the MBNL2-high subgroup exceeded that in the MBNL2-low subgroup, suggesting that elevated MBNL2 expression confers enhanced stromal communication capacity to tumor cells. Further analysis identified six ligand-receptor pairs uniquely present in the MBNL2-high subgroup, whereas only one pair (APP-CD74) was uniquely detected in the MBNL2-low subgroup. The six unique pairs in the MBNL2-high subgroup were enriched in two key signaling pathways: (1) WNT pathway-associated pairs, including WNT7B-(FZD7+LRP5), WNT9A-(FZD7+LRP5), and WNT5A-FZD7; and (2) EGF pathway-associated pairs, including TGFA-EGFR, AREG-EGFR, and HBEGF-EGFR (**Figure 7H**). To explore the communication patterns through which FAP^+^ CAFs may contribute to immunosuppression, we analyzed all inferred signals along the FAP^+^ CAF to Treg axis. A total of 26 significant ligand-receptor pairs were identified (P < 0.05). These interactions were predominantly enriched for ECM remodeling-related pathways and the MDK-NCL axis, suggesting a potential mechanism by which FAP^+^ CAFs could shape an immunosuppressive microenvironment (**Figure 7I**).

### 3.10 Validation of MBNL2 in association with response to NACI

**Figure 8:**
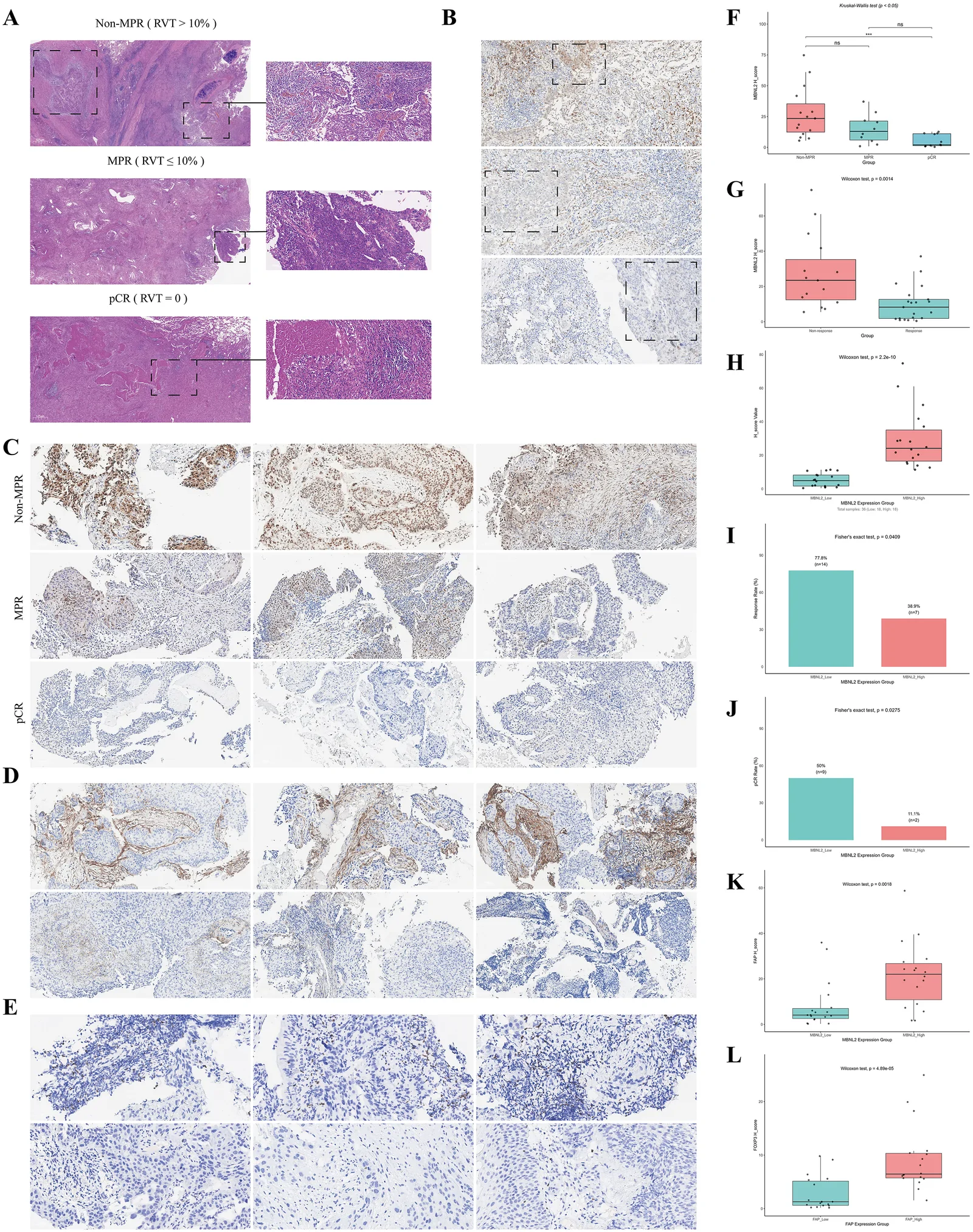
Validation of MBNL2 association with pathological response to NACI by immunohistochemistry. **(A)** Comparison of HE staining across different pathologic response grades following neoadjuvant chemoimmunotherapy. The images contrast the representative histological morphologies of Non-MPR, MPR, and pCR. **(B)** Difference in MBNL2 immunohistochemical staining between tumor and normal tissues in LUSC patients. The tumor tissue is outlined by the dashed box. **(C)** Immunohistochemical staining of MBNL2 in LUSC patients categorized as Non-MPR, MPR, and pCR. **(D)** Immunohistochemical staining of FAP in MBNL2 high- and low-expression groups. **(E)** Immunohistochemical staining of FOXP3 in FAP high- and low-expression groups. **(F)** Statistical comparison of MBNL2 immunohistochemical H-scores among LUSC patients with Non-MPR, MPR, and pCR. **(G)** Statistical comparison of MBNL2 immunohistochemical H-scores between responders and non-responders among LUSC patients. **(H)** Distribution of MBNL2 immunohistochemical H-scores in LUSC patients categorized as MBNL2 high- and low-expression groups. **(I)** Statistical comparison of pathologic response rates between MBNL2 high- and low-expression groups in LUSC patients. **(J)** Statistical comparison of pCR rates between MBNL2 high- and low-expression groups in LUSC patients. **(K)** Statistical comparison of FAP immunohistochemical H-scores between MBNL2 high- and low-expression groups in LUSC patients. **(L)** Statistical comparison of FOXP3 immunohistochemical H-scores between FAP high- and low-expression groups in LUSC patients, ***p < 0.001.

Postoperative LUSC specimens from patients who received NACI were pathologically evaluated and classified into non-MPR, MPR, or pCR groups (**Figure 8A**). Irrespective of the expression level in tumor cells, MBNL2 expression in tumor tissues was invariably weaker than in normal tissues (**Figure 8B**), consistent with the differential expression analysis (**Figure 1A**). Immunohistochemistry on preoperative biopsy specimens revealed that MBNL2 expression in tumor cells presented no significant difference between the non-MPR and MPR groups, or between MPR and pCR groups (both P > 0.05). However, expression was significantly higher in the non-MPR group compared to the pCR group (P < 0.001) and compared to the combined responder group (MPR + pCR; P = 0.0014) (**Figures 8C, F, G**). Patients with low MBNL2 expression in tumor cells had significantly higher pathological response rates (P = 0.0409) and higher pCR rates (P = 0.0275) than those with high expression (**Figures 8H–J**). These findings suggest that low MBNL2 expression in tumor cells is associated with better pathological response to NACI. Consistently, immunohistochemistry further showed that patients with high MBNL2 expression in tumor cells exhibited significantly elevated FAP expression compared to those with low MBNL2 expression (**Figures 8D, K**). Moreover, patients with high FAP expression displayed substantially elevated FOXP3 expression levels (**Figures 8E, L**).

**Figure 9:**
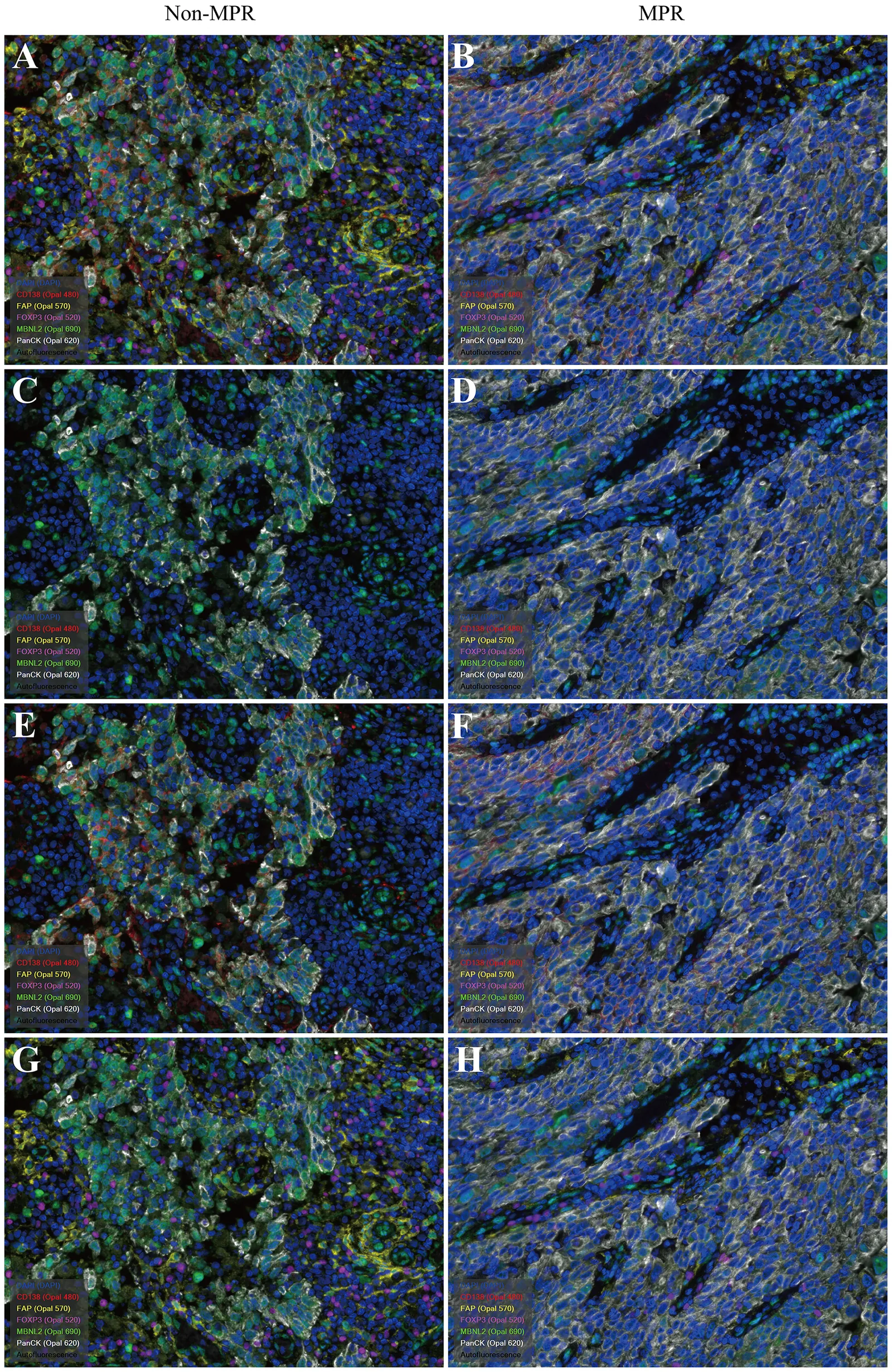
Multiplex immunofluorescence validation of MBNL2-associated cellular crosstalk in the immunosuppressive microenvironment. **(A, B)** Representative mIF staining panoramic view of two selected postoperative tissue samples (Non-MPR and MPR). **(C, D)** In mIF displaying only the DAPI, PanCK, and MBNL2 channels, the expression of MBNL2 in tumor cells was compared between Non-MPR and MPR patients. **(E, F)** In the images displaying only the DAPI, PanCK, MBNL2, and CD138 channels, MBNL2 co-localizes with the plasma cell marker CD138. **(G, H)** In the images displaying the DAPI, PanCK, MBNL2, FAP, and FOXP3 channels, the expression pattern of MBNL2 is shown, along with the spatial association between FAP^+^ CAF abundance and FOXP3^+^ regulatory T cell enrichment.

Two representative post-NACI resection specimens were selected for multiplex immunofluorescence: one MBNL2-high/non-MPR and one MBNL2-low/MPR (**Figures 9A, B**). Multiplex staining confirmed higher MBNL2 expression in tumor cells of the non-MPR case (**Figures 9C, D**). In addition, co-expression analysis revealed MBNL2 localization in plasma cells, as evidenced by MBNL2 colocalization with CD138 (**Figures 9E, F**). Notably, multiplex immunofluorescence yielded results consistent with the immunohistochemical findings: FAP^+^ CAFs were more abundant in non-MPR tumors with high MBNL2 expression in tumor cells, and regions enriched with FAP^+^ CAFs also exhibited significant accumulation of FOXP3^+^ Tregs (**Figures 9G, H**). Given that these multiplex immunofluorescence analyses were performed on two representative cases, these observations should be regarded as illustrative spatial visualization rather than quantitative validation.

Given the limited sample size of our validation cohort, we also performed exploratory univariate and multivariable logistic regression analyses to preliminarily assess the association between MBNL2 H-score and pathological response to NACI. In univariate analysis, higher MBNL2 H-score was significantly associated with lower odds of pathological response (OR = 0.916, 95% CI: 0.844–0.971, P = 0.013), indicating that patients with elevated tumor-cell MBNL2 expression were less likely to achieve MPR or pCR. Other clinical variables—including age (P = 0.192), sex (P = 0.807), tumor diameter (P = 0.760), and treatment cycles (P = 0.075)—did not reach statistical significance in univariate analysis. To further evaluate the robustness of this association, we constructed three exploratory multivariable models adjusting for different covariate combinations: (1) age and treatment cycles; (2) age and tumor diameter; and (3) treatment cycles alone (the only covariate with P < 0.1 in univariate screening). MBNL2 H-score remained nominally significant in all three models (adjusted for age and cycles: OR = 0.923, P = 0.023; adjusted for age and tumor diameter: OR = 0.908, P = 0.012; adjusted for cycles alone: OR = 0.923, P = 0.018; **Supplementary Figure 3**). It is important to note that, given the limited number of events in this cohort (n = 36, with 21 responders), we did not simultaneously include all clinical variables (age, sex, tumor diameter, and treatment cycles) in a single multivariable model. Such a fully adjusted model would suffer from severe overfitting and yield unreliable parameter estimates. Therefore, these analyses should be regarded as purely exploratory and do not establish MBNL2 as an independent predictor of pathological response; their clinical significance requires validation in larger prospective cohorts.

## 4 Discussion

In this study, through integrative multi-omics analysis, we identified MBNL2 as a previously underappreciated correlate of poor pathological response to NACI in LUSC. A 25-gene prognostic model constructed from TCGA-LUSC data accurately stratified patient survival, and the risk score remained an independent prognostic factor after adjustment for clinical variables. Given that this risk score independently reflects an immunosuppressive microenvironment characterized by T-cell dysfunction and CAF enrichment, and these features are known to be associated with poor immunotherapy outcomes (35–38), we next sought to identify genes linked to treatment response. Among them, MBNL2 was particularly prominent: patients with higher MBNL2 expression in tumor cells exhibited a significantly lower TIDE-predicted immunotherapy response rate and markedly higher CAF scores. It is important to note that TIDE is a computational prediction tool; therefore, these findings reflect inferred probabilities of response rather than confirmed clinical outcomes. The actual association between MBNL2 and treatment response was validated using pathological response data from our independent NACI cohort, where low MBNL2 expression correlated with higher pathological response and pCR rates, accompanied by corresponding changes in FAP and FOXP3 levels. Single-cell RNA-seq of post-NACI specimens further suggested enhanced ligand-receptor crosstalk between MBNL2-high tumor cells and FAP^+^ CAFs, linking tumor-intrinsic gene expression to an immunosuppressive niche. Collectively, the risk model-based nomogram represents a potential prognostic stratification tool for LUSC that warrants external prospective validation prior to clinical translation, and more importantly, we identify MBNL2 as a novel biomarker linked to NACI resistance specifically in the context of LUSC.

As a member of the muscleblind-like protein family, MBNL2 has traditionally been studied for its regulatory role in alternative RNA splicing, particularly in myotonic dystrophy and neurodevelopmental disorders (39–43). Given the conservation of splicing regulatory networks in eukaryotic cells and the general understanding that aberrant splicing events are key drivers of cancer initiation and progression (44, 45), the splicing regulatory function of MBNL2 in the nervous system suggests that it may also play an important role in tumorigenesis. Indeed, in recent years, a growing number of studies have begun to focus on the aberrant expression of MBNL2 and its prognostic value in solid tumors, though the findings reveal a context-dependent functional duality. In hepatocellular carcinoma, MBNL2 overexpression was paradoxically associated with smaller tumor size, lower tumor stage, and a trend toward better overall survival, and functional experiments confirmed its tumor-suppressive role through inhibition of proliferation, migration, and invasion (46). Similarly, in breast and liver cancers, MBNL2 was found to be downregulated and to suppress metastasis via the miR-182–MBNL2–AKT–EMT signaling axis (47). In contrast, other malignancies exhibit an opposing pattern: in esophageal squamous cell carcinoma, MBNL2 is overexpressed and promotes migration and invasion through EMT facilitation, correlating with advanced tumor stage and poor prognosis (48). Furthermore, in ovarian cancer, elevated MBNL2 expression was linked to cisplatin resistance and shorter relapse-free survival, with lower expression predicting better chemotherapy response (49). These conflicting observations suggest that MBNL2 may exert either tumor-suppressive or tumor-promoting functions depending on the specific tissue context and molecular milieu. However, its biological role in LUSC, particularly its involvement in the tumor immune microenvironment, has remained largely unexplored.

To explore this, functional enrichment and GEPIA analyses revealed that MBNL2-associated genes are involved in ECM organization, and that MBNL2 expression correlates with FAP and FOXP3 levels. Together with the positive association between MBNL2 expression and TIDE-derived CAF and dysfunction scores, these integrative findings suggest that MBNL2 may contribute to an immunosuppressive microenvironment through ECM remodeling and CAF activation. This was corroborated by IHC validation in pre-NACI biopsies. CellChat analysis of post-treatment scRNA-seq data further suggested that MBNL2-high tumor cells exhibit enhanced communication with FAP^+^ CAFs, primarily through WNT and EGF pathway ligands. With respect to the WNT axis, studies in colorectal cancer have demonstrated that Wnt signaling directly promotes fibroblast activation and contraction and regulates CAF phenotypic plasticity (50–52). This computationally predicted WNT-mediated crosstalk raises the hypothesis that it may contribute to a pro-tumorigenic CAF state in LUSC, providing a testable lead for future mechanistic studies. Along the EGF axis, AREG-EGFR was specifically enriched among the unique pairs. Prior work has shown that IL1RL1^+^ Tregs engage CAFs through the AREG-EGFR signaling axis, driving a pro-fibrotic and immunosuppressive CAF phenotype that poses a key barrier to effective immunotherapy (53). The presence of this same ligand-receptor pair in the tumor-CAF communication network raises the hypothesis that MBNL2-high tumor cells may functionally mimic Tregs via AREG-EGFR. The specific enrichment of the AREG-EGFR axis is linked to an altered CAF status and an immunosuppressive microenvironment, features that accompany NACI resistance in our cohort. Functional studies are needed to test this potential mechanistic link. In hepatocellular carcinoma, Scissor^+^ malignant epithelial cells were shown to recruit CD4^+^ Tregs via CCL20-CCR6 and enrich BCAM^+^ CAFs through MDK-NCL, thereby remodeling the metastatic microenvironment (54). Separately, CEBPB^+^ CAFs were found to communicate with T cells via MDK-NCL, and with B cells and myeloid cells through MDK-SDC1 and other pathways (55). Consistent with this paradigm, CellChat analysis along the FAP^+^ CAF to Treg axis revealed that inferred FAP^+^ CAF–Treg communications are primarily enriched for ECM remodeling-related ligand-receptor pairs and the MDK-NCL axis. These computationally predicted interactions are features consistent with the assembly of an immunosuppressive niche. This convergence identifies MDK-NCL as a candidate axis that, based on our predictive analyses, may be involved in Treg recruitment and immunosuppression; its actual biological role warrants dedicated investigation. Multiplex immunofluorescence performed on post-NACI resection specimens further visualized the spatial co-localization and enrichment of FAP^+^ CAFs and FOXP3^+^ Tregs, revealing a tendency toward their co-enrichment in MBNL2-high tumors with poor response, thereby providing morphological support for the computationally inferred tumor-stromal communication network. Despite the limitation that single-cell data were derived from post-treatment specimens, convergent findings from multiple independent analytical layers support an association between MBNL2 and NACI resistance. Our pre-treatment IHC data demonstrated a significant correlation between lower tumor-cell MBNL2 expression and higher pathological response rates. Independently, TCGA-LUSC data from treatment-naïve tumors showed that MBNL2 expression positively correlates with CAF abundance and T-cell dysfunction scores. The CellChat-inferred communication patterns in post-NACI residual tumors—enhanced WNT/EGF crosstalk with FAP^+^ CAFs and MDK-NCL-mediated Treg interactions—while not distinguishable as cause or consequence of resistance, are consistent with the immunosuppressive features associated with MBNL2-high tumors in both IHC and TCGA cohorts. The dual advantage in “quantity” (increased average probability) and “quality” (acquisition of unique signaling in key pathways) observed in the MBNL2-high tumor cell group provides a plausible mechanistic framework for the negative correlation between high MBNL2 expression and poor immunotherapy response observed across our analyses. Taken together, this multi-layered convergence supports the biological plausibility of MBNL2 as a correlate of poor NACI response and positions it as a novel biomarker linked to CAF activation in LUSC, while underscoring the need for prospective studies with paired pre- and post-treatment specimens to establish mechanistic causality.

Notably, although the overall expression level of MBNL2 in LUSC tumor tissues was lower than that in normal lung tissues, significant heterogeneity in MBNL2 expression was observed among cancer cells (**Figure 7E**). Our study revealed that this intercellular variability in MBNL2 expression within cancer cells was significantly associated with the activation status of CAFs in the TME and resistance to immunotherapy (**Figures 7G–I**). This suggests that, even in the context of overall low expression, relatively high MBNL2 expression in cancer cells is strongly associated with microenvironmental remodeling. We hypothesize that high MBNL2 expression in normal lung tissue may help maintain lung tissue homeostasis. Conversely, within tumors, the ‘aberrant upregulation’ of MBNL2 in a subset of cancer cells correlates with stromal remodeling programs and an immunosuppressive niche, which are observed in parallel with immunotherapy resistance. Whether MBNL2 directly drives these changes remains to be determined.

Although this study has revealed the significance of MBNL2 in immunotherapy resistance in LUSC through multi-omics integration and histopathological validation, several limitations remain. First, both the 25-gene prognostic model and the MBNL2-NACI response correlation derive from retrospective data and lack prospective, multicenter, randomized validation. Moreover, the clinical validation cohort is relatively small (n=36), and subgroup analyses by pathological response (e.g., non-MPR, MPR, pCR) are constrained by even smaller patient numbers. Owing to this limited sample size, multivariate analysis to determine whether MBNL2 expression is an independent predictor of pathological response beyond other clinical variables could not be reliably performed. Accordingly, the clinical significance of these findings should be interpreted with caution until confirmed in larger, independent cohorts. The limited sample size partly reflects the stringent inclusion criteria: all patients were required to have pre-NACI biopsy specimens obtained and diagnosed at our center, with complete post-surgical pathological response data — a combination that is inherently difficult to accrue in a single-center retrospective setting. Second, the enhanced WNT and EGF pathway crosstalk between MBNL2-high tumor cells and FAP^+^ CAFs inferred by CellChat remains a bioinformatic prediction and requires functional validation through gain- and loss-of-function experiments in LUSC cell lines, co-culture systems, and animal models. Third, owing to the scarcity of untreated pre-NACI LUSC specimens with matched pathological response data, single-cell analysis was performed on post-NACI resection samples. Consequently, the CellChat-inferred communication patterns between MBNL2-high tumor cells and FAP^+^ CAFs cannot be definitively distinguished as a cause or consequence of NACI resistance, as they may partly reflect treatment-induced stromal remodeling rather than pre-existing resistance mechanisms. Nevertheless, the convergent evidence from treatment-naïve TCGA-LUSC data—showing positive correlations between MBNL2 expression, CAF abundance, and T-cell dysfunction scores—provides independent support for an intrinsic association between MBNL2 and an immunosuppressive microenvironment that is not contingent upon NACI treatment. This mitigates, though does not eliminate, the concern that the observed communication patterns are entirely therapy-induced. Future studies employing paired pre- and post-treatment single-cell profiling are essential to disentangle pre-existing resistance mechanisms from treatment-elicited stromal changes. Additionally, the multiplex immunofluorescence analysis was limited to two representative cases and serves only as an illustrative proof-of-concept; quantitative validation in a larger cohort is needed to confirm the spatial relationships observed. Moreover, although scRNA-seq revealed abundant MBNL2 expression in other cell types such as plasma cells, this study focused on tumor-cell MBNL2, leaving its independent roles and intercellular crosstalk in other compartments largely unexplored, which limits a comprehensive view of its multifaceted TME functions. Finally, the upstream mechanisms driving heterogeneous MBNL2 expression — such as promoter methylation, a hypoxic microenvironment, or non-coding RNA networks — were not investigated. Future research should integrate cell-specific knockout animal models, larger cohorts with single-cell resolution analysis, and clinical samples from uniform treatment regimens, aiming to translate MBNL2 from a correlative marker into a viable interventional target.

In summary, through multi-omics integration and histopathological validation, this study establishes a 25-gene prognostic model for LUSC and, more importantly, uncovers MBNL2 as a previously unrecognized correlate of poor pathological response to NACI. Our findings demonstrate a robust correlation between elevated MBNL2 expression in tumor cells and enhanced tumor-CAF crosstalk and CAF activation. These features co-occur with an immunosuppressive microenvironment and reduced immunotherapy response. While these associations are strong and consistent, functional validation is now required to establish a causal role for MBNL2 in driving these processes. These observations position MBNL2 as a promising biomarker for stratifying NACI response in LUSC and provide a foundation for future mechanistic studies. Further elucidation of the mechanisms linking MBNL2 to tumor-CAF communication and the upstream drivers of its aberrant expression may inform novel therapeutic strategies aimed at overcoming immunotherapy resistance in this disease.

## Data Availability

The original contributions presented in the study are included in the article/**Supplementary Material**. Further inquiries can be directed to the corresponding authors.

## Glossary

LUSC: lung squamous cell carcinoma
NSCLC: non-small cell lung cancer
LUAD: lung adenocarcinomas
ICIs: immune checkpoint inhibitors
PD-1: programmed death-1
PD-L1: programmed death-ligand 1
EFS: event-free survival
pCR: pathological complete response
NCAI: neoadjuvant chemoimmunotherapy
RVT: residual viable tumor
MPR: major pathological response
Non-MPR: non-major pathological response
TMB: tumor mutational burden
TPS: tumor proportion score
TME: tumor microenvironment
CAFs: cancer-associated fibroblasts
FAP: fibroblast activation protein
ECM: extracellular matrix
Tregs: regulatory T cells
TCGA: The Cancer Genome Atlas
LASSO: least absolute shrinkage and selection operator
MBNL2: muscleblind-like protein 2
TIDE: Tumor Immune Dysfunction and Exclusion
IHC: immunohistochemistry
mIF: multiplex immunofluorescence
FDR: false discovery rate
DEGs: differentially expressed genes
ROC: receiver operating characteristic
AUC: area under the curve
GBM: gradient boosting machine
RF: random forest
SVM: support vector machine
ssGSEA: Single-sample gene set enrichment analysis
GO: gene ontology
BP: biological process
CC: cellular component
MF: molecular function
KEGG: kyoto encyclopedia of genes and genomes
PCA: Principal component analysis
UMAP: uniform manifold approximation and projection
OS: overall survival
HR: hazard ratio
EMT: epithelial-mesenchymal transition.
