# Supplementary material for: Multi-omics identification of MBNL2 associated with poor pathological response to neoadjuvant chemoimmunotherapy in lung squamous cell carcinoma
Source: Front Oncol. 2026 Aug 19;16:1877829. doi: 10.3389/fonc.2026.1877829 (PMC13533729; doi:10.3389/fonc.2026.1877829)
Supplement: Supplementary file 1 [file DataSheet1.zip › 0720-Supplementary_Material.docx]

Supplementary Material

**Supplementary** **Table 1**

The genes and coefficients from the LASSO model

| Gene | Coefficient |
| --- | --- |
| ADAMTS17 | -0.161896195367618 |
| FAM25A | 1.02172762788371 |
| SDHAF3 | -0.0530530210813457 |
| LYSMD1 | -0.105378002873874 |
| SERPIND1 | 0.00219551933128211 |
| ELMO3 | 0.202347324813373 |
| UGT2B11 | 0.324707130144543 |
| DPPA2 | 0.136799086733475 |
| F13A1 | 0.0595693038222112 |
| PCDHA3 | 0.785710796579667 |
| TRIM58 | 0.173269923769442 |
| MBNL2 | 0.0728119923768193 |
| WFS1 | 0.0670872488608741 |
| LCE3C | 0.292576586753664 |
| RGS3 | -0.0267716602929376 |
| CHRDL1 | 0.034750395878724 |
| SERPINB13 | -0.0157145558311031 |
| CALB1 | -0.0622361889588651 |
| HRASLS | -0.0711728073963037 |
| STXBP1 | 0.0820444950618767 |
| LBP | 0.0994436713644892 |
| IER5L | 0.0744989591433282 |
| SLC28A1 | 0.563776223096105 |
| CYP19A1 | 0.476630044826396 |
| MMRN1 | 0.0295441532693364 |

**Supplementary Figure 1.** Flow diagram of the study.

**Supplementary Figure 2.** Annotation of major cell types and characterization of epithelial and T cell subpopulations. (A) The expression of PTPRC, CD3E, LYZ, CD79A, MS4A1, IGHG1, CSF3R, KIT, VWF, COL1A1, EPCAM, and MKI67 in the 23 clustered cell populations. (B) Differentially expressed genes in each epithelial subclusters were ranked by average log2 fold change, and the top 5 marker genes are shown. (C) Heatmap visualization of epithelial cells classified as aneuploid (malignant) or diploid (normal) by the CopyKAT algorithm. (D) The expression of CD4, CD8A, CD8B, GZMK, GZMB, FGFBP2, FCGR3A, FOXP3, IL2RA, and MKI67 in the 8 T cell subsets. (E) Differentially expressed genes in each T cell subclusters were ranked by average log2 fold change, and the top 5 marker genes are shown.

**Supplementary Figure 3.** Exploratory logistic regression analysis of MBNL2 H-score and pathological response in the validation cohort. Forest plot showing odds ratios (OR) and 95% confidence intervals from univariate and exploratory multivariable models. OR < 1 indicates lower odds of pathological response (MPR or pCR) with higher MBNL2 H-score. P values are shown in parentheses next to each model name. Given the limited sample size (n=36, 21 events), these analyses are exploratory and should not be interpreted as definitive evidence of independent prediction.
